# SIPGCN: A Novel Deep Learning Model for Predicting Self-Interacting Proteins from Sequence Information Using Graph Convolutional Networks

**DOI:** 10.3390/biomedicines10071543

**Published:** 2022-06-29

**Authors:** Ying Wang, Lin-Lin Wang, Leon Wong, Yang Li, Lei Wang, Zhu-Hong You

**Affiliations:** 1 College of Information Science and Engineering, Zaozhuang University, Zaozhuang 277160, China; zzxywy@uzz.edu.cn; 2 Big Data and Intelligent Computing Research Center, Guangxi Academy of Sciences, Nanning 530007, China; lghuang@gxas.cn (L.W.); zhuhongyou@gmail.com (Z.-H.Y.); 3 School of Computer Science and Information Engineering, Hefei University of Technology, Hefei 230601, China; 2021010123@mail.hfut.edu.cn; 4 School of Computer Science, Northwestern Polytechnical University, Xi’an 710129, China

**Keywords:** self-interacting protein, graph convolutional networks, protein–protein interactions, random forest

## Abstract

Protein is the basic organic substance that constitutes the cell and is the material condition for the life activity and the guarantee of the biological function activity. Elucidating the interactions and functions of proteins is a central task in exploring the mysteries of life. As an important protein interaction, self-interacting protein (SIP) has a critical role. The fast growth of high-throughput experimental techniques among biomolecules has led to a massive influx of available SIP data. How to conduct scientific research using the massive amount of SIP data has become a new challenge that is being faced in related research fields such as biology and medicine. In this work, we design an SIP prediction method SIPGCN using a deep learning graph convolutional network (GCN) based on protein sequences. First, protein sequences are characterized using a position-specific scoring matrix, which is able to describe the biological evolutionary message, then their hidden features are extracted by the deep learning method GCN, and, finally, the random forest is utilized to predict whether there are interrelationships between proteins. In the cross-validation experiment, SIPGCN achieved 93.65% accuracy and 99.64% specificity in the human data set. SIPGCN achieved 90.69% and 99.08% of these two indicators in the yeast data set, respectively. Compared with other feature models and previous methods, SIPGCN showed excellent results. These outcomes suggest that SIPGCN may be a suitable instrument for predicting SIP and may be a reliable candidate for future wet experiments.

## 1. Introduction

Protein is the basic component of organisms and participates in almost all biological processes in cells [1,2]. The vast majority of life activities are the result of the simultaneous action of many proteins, and the interacting protein system is the basis for all life activities. Exploring the interaction between proteins (PPIs) is not only of great significance to the regulation of cell growth, but also lays a theoretical foundation for deeper disease research [3,4,5,6]. With the fast growth of high-throughput experimental techniques for measuring the interactions between organisms, massive amounts of experimental data on various types of proteins continue to accumulate. This makes it possible to develop effective new theories of analysis and computation that can contribute to a deeper understanding of the mechanisms through which cellular functions arise, providing useful information for studies such as the discovery of evolutionary patterns and even the pathogenic mechanisms of organisms.

In the interaction between proteins, self-interacting protein (SIP) accounts for a large proportion. SIP is an interaction between the same type of proteins, which holds a crucial position as a coordinator in gene expression and other complex biological functions. In-depth studies can help to better understand cancer, viral infections, neurodevelopmental disorders, and other potential diseases disturbed by these factors [7,8,9,10]. In their study of the origin of protein evolution, Pereira Leal et al. [11] discovered that proteins evolved through the replication of homodimers, in other words, through the replication of genes encoding SIPs. Wagner et al. [12] found, in fly, worm, and yeast data sets, that the PPIs between paralogous dimers and homodimers are not independent, and the frequency of the protein–protein interactions between paralogous proteins are higher than that of pure chance. Thus, they believe that gene differentiation and replication are important factors to promote the eukaryotic proteome expansion, and a multicopy of the same subunit is an economic way to form a larger functional structure. Ispolatov et al. [13] identified a significant amount of SIP in the PPI network, which suggests that it may play a vital function in the cellular system. Moreover, SIP is able to regulate protein function without increasing the genome size, thus expanding its functional diversity.

With the deepening of computational biology research and the accumulation of experimental data [14,15,16], the computational methods of protein–protein interaction prediction are emerging, and excellent results have been achieved [10,17,18,19,20]. Among them, the neural network plays an important role. It has a self-learning ability and associative storage function. It can learn the distribution rule of original biological data and find the optimal solution of a computational problem at high speed, so as to effectively improve the performance of the model. For instance, Wang et al. [21] designed the CNNFSRF method to accurately predict PPI by extracting the features of the protein sequences through convolutional neural networks. In order to effectively combine the key sequence-pattern and sequence-order information when processing the protein samples, Jia et al. [22] used chaos game to represent information for predicting PPI. You et al. [23] designed a hierarchical PPI prediction model, PCAEELM, based on protein sequences only. The model refines the protein data features using the PCA algorithm, and is combined with a high-performance ELM classifier to achieve a high accuracy. Chen et al. [24] combined the location-specific score matrix with wavelet transform to extract protein features, and effectively predicted SIPs using the deep forest-based predictor. In both human and yeast data sets, the proposed model achieves competitive accuracy. Wang et al. [25] designed the computing method to predict PPI based on the similarity between protein sequences and natural language, and achieved a better performance in the cross-validation experiments. Liu et al. [26] proposed an SIP prediction computational approach called SPAR, which considers fine-grained domain interaction information to design an improved coding scheme. Compared with other SIP prediction models, this method shows a better performance.

**Figure 1:**
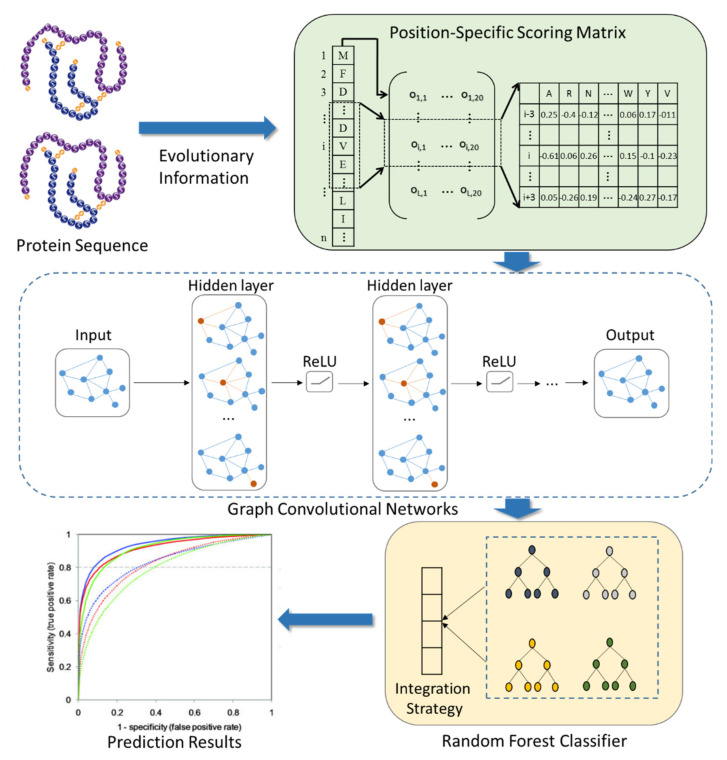
The flowchart of SIPGCN.

In this work, we combine a deep learning graph convolutional neural network with protein sequences to design the SIP prediction model, SIPGCN. To be specific, SIPGCN first characterizes the biological evolutionary message of the protein amino acid by the PSSM matrix, then extracts their objective distribution features using GCN, and finally predicts the existence of interacting SIPs using an RF classifier. In the human data set, SIPGCN obtained an accuracy of 93.65% and a specificity of 99.64%. In the yeast data set, SIPGCN achieved 90.69% and 99.08% for these evaluation metrics, respectively. Compared with the previous models, SIPGCN exhibited superior competitiveness. The outcomes of these experiments demonstrate that SIPGCN can effectively predict SIPs with interactions from massive quantities of data and can provide reliable candidates for subsequent wet experiments, the flowchart of which is illustrated in Figure 1. The source code and data used by SIPGCN can be downloaded from https://github.com/look0012/SIPGCN (accessed on 25 May 2022).

## 2. Materials and Methods

### 2.1. Gold Standard Data Sources

In this experiment, we collected two identical proteins from the relevant databases and their interaction mode was described as “direct interaction” to construct the experimental data set. More concretely, human protein sequences with self-interrelation were collated from databases including InnateDB [27], BioGRID [28], UniProt [29], DIP [30], and MatrixDB [31]. The principles for selecting these data are as follows: Firstly, the length of protein residues was 50 to 5000 residues. Secondly, only proteins that met one of the following conditions could be selected as positive samples: (1) officially reported by two or more journals, (2) proteins defined as homo-oligomers by the UniProt database, and (3) verified by more than two large-scale or one small-scale experiments. Finally, the negative samples did not contain proteins with self-interaction. Through screening of the above principles, 1441 SIPs and 15,938 non-SIPs were included in the human data set. Additionally, the yeast data set also underwent the same screening, and the quantity of positive and negative samples was 710 and 5511, respectively.

In this study, the data set we used was imbalanced, with the number of negative samples being much larger than the number of positive samples. Generally speaking, the vast majority of data sets in the real world are imbalanced. We would be very lucky if we could obtain a balanced data set. Therefore, to solve the problem of imbalanced data, researchers have put forward many solutions, which can be roughly divided into two categories: one is to build balanced data sets and the other is to use different evaluation indicators to measure the imbalanced data sets.

For the first scheme, we used the resampling method. For example, we used the over-sampling method to increase the number of minority class samples to the same number as that of the majority classes. Another is the use of the undersampling method to select part of the majority class samples to reduce them to the same number as the minority class samples. In addition, the data set can be balanced by generating more virtual samples through GAN and other methods.

For the second scheme, accuracy was not a good measure. It was more inclined to the majority of samples, which is often misleading. Therefore, in addition to accuracy, some other evaluation indicators need to be added to measure the performance of the model. For example, a comprehensive evaluation index F1 that can reflect the accuracy and recall rate, namely, an AUC that can consider the classification ability of the classifier for positive and negative samples at the same time, and can still make a reasonable evaluation of the classifier in the case of unbalanced samples.

In this study, the gold standard data set we used produced both positive and negative samples. Unlike some data sets that only produced positive samples, we needed to build negative samples (in this case, we built a balanced data set). Based on the consideration of maintaining the integrity of the data set, we did not delete the samples of the data set, but used all of the samples of the imbalanced data set. Therefore, in addition to using accuracy, we also used some more reliable measures, such as F1, MCC, and AUC, and drew the ROC curve. We used these comprehensive indicators to better evaluate the performance of the model.

### 2.2. Characterization of Protein Evolution Information

We utilized the PSSM matrix to transform the protein evolution information in alphabetic form into a matrix in numerical form in the experiment. PSSM [32] is able to translate protein sequences into numerical matrices and depict their biological evolutionary information [33,34,35,36,37]. In the PSSM matrix, each protein can generate a N×20 matrix$\;PM\left(i,j\right)$, which is mathematically described below:

(1) $$PM=\left[\begin{array}{cccc} \sigma_{1,1} & \sigma_{1,2} & \cdots & \sigma_{1,20}\\ \sigma_{2,1} & \sigma_{2,2} & \cdots & \sigma_{2,20}\\ \vdots \; & \vdots \; & \vdots \;\;\; & \vdots \\ \sigma_{N,1} & \sigma_{N,2} & \cdots & \sigma_{N,20} \end{array}\right]$$

here, N means the quantity of protein residues, 20 means the quantity of amino acid types, and the matrix element $\sigma_{i,j}$ denotes the probability of mutation of the ith residue to the *j*th amino acid. In the experiment, we used position-specific iterated BLAST (PSI-BLAST) to generate the PSSM matrix of protein, and its download website is http://blast.ncbi.nlm.nih.gov/blast.cgi (accessed date 1 May 2015). We set the parameter e-value and iterations of PSI-BLAST to the optimal 0.001 and 3, respectively, and searched for the protein sequences in the classical SwissProt database.

### 2.3. Protein Feature Extraction

In the experiment, Fast learning with Graph Convolutional Networks (FastGCN) is employed to extract the hidden features of the proteins [38]. FastGCN is able to interpret graph nodes as independent identically distributed samples under a certain probability distribution and write the loss and each convolutional layer as an integral over the vertex embedding function, and to then evaluate the integral by defining a Monte Carlo approximation to the sample loss and sample gradient.

Suppose the probability space $\left(V^{\prime },F,P\right)$ correlates with the vertex set $V^{\prime }$ of graph $G^{\prime }$. For a subgraph G of a graph $G^{\prime }$, its vertices are i.i.d. samples of $V^{\prime }$ obtained by the probability measure P. It is mathematically represented as follows.

(2) $${\tilde{h}}^{\left(l+1\right)}\left(v\right)=\int \hat{A}\left(v,u\right)h^{\left(l\right)}\left(u\right)W^{\left(l\right)}dP\left(u\right),\;\;\;h^{\left(l+1\right)}\left(v\right)=\sigma \left({\tilde{h}}^{\left(l+1\right)}\left(v\right)\right),\;\;\;l=0,\ldots ,M-1$$

where u and v are independent random variables of P, and $h^{\left(l\right)}$ is the embedding function from the lth layer. Loss L is the expected value of $g\left(h^{\left(M\right)}\right)$ embedded in $h^{\left(M\right)}$, which is expressed as follows:

(3) $$L=E_{v\sim P}\left[g\left(h^{\left(M\right)}\right)\left(v\right)\right]=\int g\left(h^{\left(M\right)}\right)\left(v\right)dP\left(v\right)$$

Thus, the i.i.d. sample $u_{1}^{\left(l\right)},\ldots ,u_{t_{1}}^{\left(l\right)}\sim P$ of $t_{l}$ is available to approximately estimate the integral transformation in the lth layer, which is described below:

(4) $${\tilde{h}}_{t_{l+1}}^{\left(l+1\right)}\left(v\right)≔\frac{1}{t}\sum_{j=1}^{t_{l}}\hat{A}\left(v,u_{j}^{\left(l\right)}\right)h_{t_{l}}^{\left(l\right)}\left(u_{j}^{\left(l\right)}\right)W^{\left(l\right)},\;\;\;\;h_{t_{l+1}}^{\left(l+1\right)}\left(v\right)≔\sigma \left({\tilde{h}}_{t_{l+1}}^{\left(l+1\right)}\left(v\right)\right)$$

where $h_{t_{0}}^{\left(0\right)}$ is $h^{\left(0\right)}$. The loss L is translatable to the following:

(5) $$L_{t_{0},t_{1},\ldots ,t_{M}}≔\frac{1}{t_{M}}\sum_{i=1}^{t_{M}}g\left(h_{t_{M}}^{\left(M\right)}\left(u_{i}^{\left(M\right)}\right)\right)$$

In the experiment, we verified the hyperparameter of FastGCN through the grid search method, and its optimization setting was as follows: the learning rate was 1e-1, the number of hidden layer neurons was 256, the number of iterations was 200, and the loss function was thr L2 regularization function. Specific experimental details can be found in Supplementary Materials Table S1.

### 2.4. Interaction Prediction

We use a random forest (RF) classifier [39,40,41] in the study to predict the interaction of the extracted feature data. RF contains multiple decision trees that classify new data by what they have learned in the data set using the following classification strategy.

(a) Construct sub-datasets by drawing samples from the dataset in a repeatable form according to the number of samples;
(b) Train decision trees based on these sub-datasets and obtain the results of each decision tree;
(c) Combine the results of all decision trees to obtain the final output using a minority–majority voting strategy.

## 3. Results

### 3.1. Evaluation Metrics

We utilize evaluation metrics commonly used in machine learning to evaluate the performance of the model in the study in order to make it generalizable and easily comparable with other methods [24,42,43,44]. These evaluation metrics can be mathematically formulated as follows:

(6) $$Acc.=\frac{TP+TN}{TP+TN+FP+FN}$$

(7) $$Spe.=\frac{TN}{TN+FP}\;$$

(8) $$F1=\frac{2TP}{2TP+FP+FN}$$

(9) $$MCC=\frac{TP\times TN-FP\times FN}{\sqrt{\left(TP+FP\right)\times \left(TN+FN\right)\times \left(TP+FN\right)\times \left(TN+FP\right)}}$$

here, *TP* and *TN* denote true positive and negative, and *FP* and *FN* denote false positive and negative, respectively. In the experiments, we also simultaneously plotted the receiver operating curve (ROC) curves and calculated the area under the curve (AUC) values to comprehensively evaluate the model capability.

Five-fold cross-validation (FFCV) [14,45,46,47] was used to generate the above evaluation criteria when evaluating the model performance. Specifically, we scrambled the order of all the data in SIPs data set, and randomly generated five disjoint subsets with an approximately equal number. In each experiment, one subset was utilized to verify the model performance, while the rest of the subsets were utilized for training the model. The experiment was run five times, and different subsets were taken each time to ensure that all subsets were verified only once. The final results were expressed by the average and standard deviation of the five groups of experiments. To minimize the effect of randomness on the assessment method, we performed 100 groups of FFCV and took the mean value as the final result.

### 3.2. Performance Evaluation

**Figure 2:**
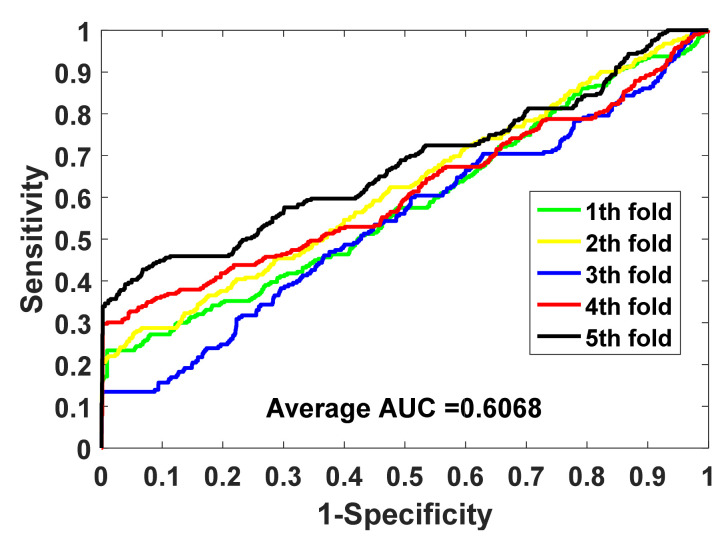
The ROC generated by SIPGCN in the human data set.

**Figure 3:**
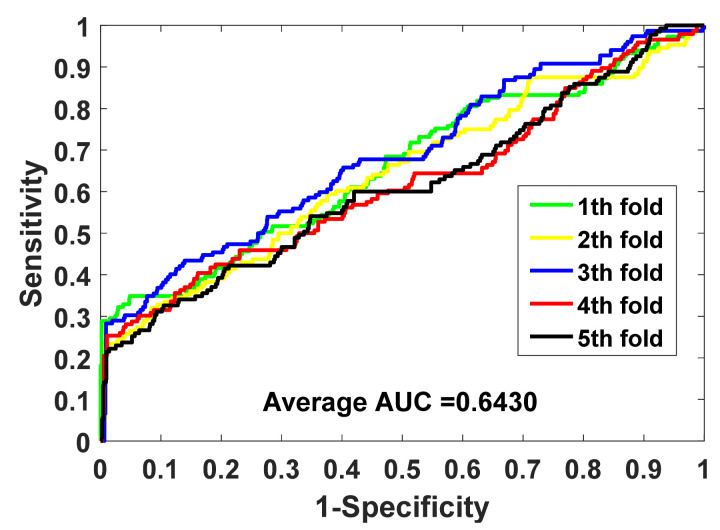
The ROC generated by SIPGCN in the yeast data set.

**Figure 4:**
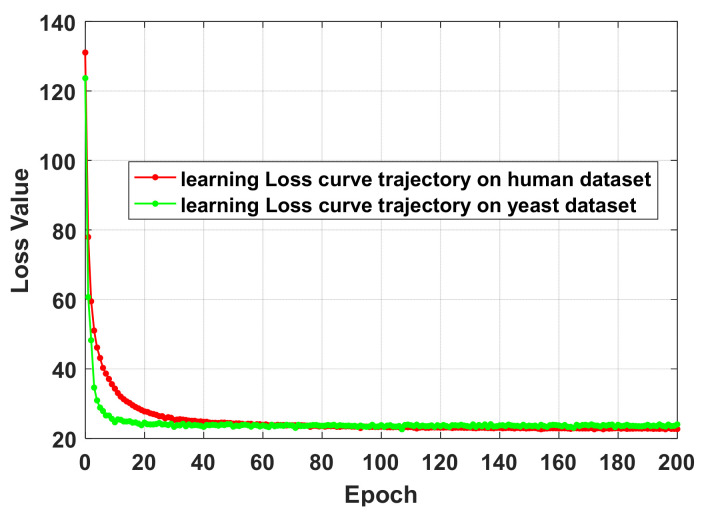
Learning curve trajectory generated by SIPGCN in the human and yeast data sets.

SIPGCN is evaluated for its performance on data sets human and yeast using the FFCV method. The detailed FFCV outcomes are summarized in Table 1 and Table 2. As seen in Table 1 of the human data set, the accuracy achieved by the five experiments was 93.53%, 93.41%, 92.78%, 94.10%, and 94.42%, with an average of 93.65% and standard variance of 0.64%. SIPGCN achieved 99.64%, 37.11%, 43.01%, and 0.6068 in specificity, F1, MCC, and AUC, respectively. As seen in Table 2, which shows the outcomes of the yeast data set, the accuracy achieved by the five SIPGCN experiments was 91.32%, 91.08%, 90.35%, 90.11%, and 90.60%, with an average of 90.69% and a standard variance of 0.50%. Among other evaluation indicators, SIPGCN achieved 99.08%, 38.37%, 41.19%, and 0.6430. The ROC on gold standard data sets are displayed in Figure 2 and Figure 3. In order to ensure that all aspects of the ML process are fully addressed and reported, so as to better evaluate the model’s capabilities, we plotted the learning curve trajectory of the model during training, as shown in Figure 4. As can be seen from the figure, the model shows a convergence trend with the increase in iteration.

### 3.3. Comparison with Other Classifier Models

To verify the effect of the classifier on the model capability, we implemented ablation experiments. In particular, we retained the feature extraction method in the experiments and only replaced the RF classifiers used in the original model with K-Nearest Neighbor (KNN) [48] and Extreme Learning Machine (ELM) [49], and validated them in human and yeast data sets, respectively, and the experimental outcomes are summarized in Table 3 and Table 4.

Table 3 lists the FFCV outcomes of the ELM and KNN classifier methods, respectively, in the human data set. We can see that the accuracy and specificity achieved by the five groups of experiments of the ELM classifier model are 87.19% and 93.26%, respectively, with a standard deviation of 0.63 and 0.68%. The average accuracy and specificity achieved by the KNN classifier model of the five groups of experiments are 87.20% and 93.31%, respectively, with a standard deviation of 0.53 and 0.48%, respectively. However, SIPGCN acquired an accuracy of 93.65% in the human data set, which is 6.46 and 6.45% higher, and a specificity of 99.64, which is 12.44 and 6.33% higher, respectively.

Table 4 lists the FFCV results of the ELM and KNN classifier models, respectively, in the yeast data set. We can see from the table that the average accuracy and specificity achieved by the five groups of experiments of the ELM classifier model are 87.19% and 93.26%, respectively, with a standard deviation of 0.63 and 0.68%, respectively. The average accuracy and specificity achieved by the KNN classifier model of the five groups of experiments are 87.20% and 93.31%, respectively, with a standard deviation of 0.53 and 0.48%, respectively. However, SIPGCN acquired an accuracy of 93.65% in the human data set, which is 6.46 and 6.45% higher, and a specificity of 99.64, which is 12.44 and 6.33% higher, respectively.

**Figure 5:**
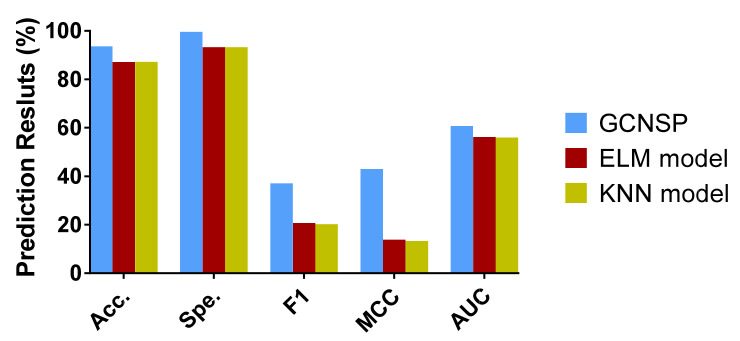
The outcomes of different classifier models in the human data set.

**Figure 6:**
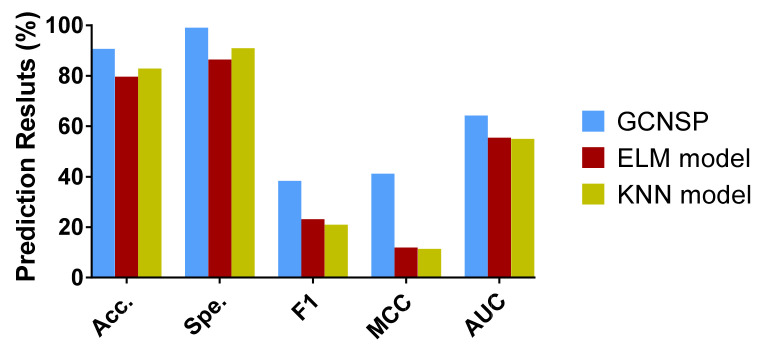
The outcomes of different classifier models in the yeast data set.

Table 4 gives a summary of the FFCV outcomes of the ELM and KNN classifier models on the yeast data set. We can see that the accuracy and specificity achieved by the five groups of experiments for the ELM classifier model are 79.68% and 86.48%, respectively, with a standard deviation of 0.94 and 0.50%, respectively. The average accuracy and specificity achieved by the KNN classifier model for the five groups of experiments are 82.86% and 90.96%, respectively, with a standard deviation of 0.87 and 0.76%, respectively. However, SIPGCN acquired an accuracy of 90.69% on the yeast data set, which is 11.01 and 7.83% higher, and a specificity of 99.08%, which is 16.22% and 8.12% higher, respectively. To observe the experimental results more intuitively, we present these evaluation indicators with histograms, as displayed in Figure 5 and Figure 6.

### 3.4. Comparison with Other Feature Models

To verify the influence of GCN features on the model performance, we compared it with the autocovariance (AC) features. Specifically, we utilized the AC method to extract protein features to replace GCN features, while the other algorithms of the model remained constant. The outcomes obtained by the AC feature model on the gold standard data sets for human and yeast are listed in Table 5 and Table 6. From Table 5, it is evident that the AC feature model gained a mean accuracy of 84.31%, and the accuracy of the FFCV was 84.12%, 83.94%, 83.22%, 85.04%, and 85.23%, respectively. The SIPGCN model achieved an accuracy of 93.65%, which is 9.34% higher than the AC feature model. Among the other parameters of the evaluation model, the SIPGCN model also achieved better results.

The outcomes of the AC feature model for the yeast dataset are summarized in Table 6, from which it can be seen that the AC feature model gained an average accuracy of 79.41%, sensitivity of 86.99%, and AUC of 55.37%. The SIPGCN model is 11.28%, 12.09%, and 8.93% higher, respectively, than the AC feature model for these parameters. From the above comparison, it is evident that SIPGCN utilizing the GCN algorithm has better outcomes than the AC feature model. This finding suggests that, compared with the AC feature model, SIPGCN has a better performance. The reason for this result may be that the GCN algorithm can deeply dig out the essential characteristics of the proteins in the form of graph structures, which helps the classifier to better identify potential protein self-interactions.

### 3.5. Comparison with Other Previous Models

Recent investigations have shown that many researchers use a convolutional network or graph convolutional neural network [50] combined with the 3D structure information of proteins to solve the problem of PPI prediction [51]. In the model evaluation, these methods have achieved good results. Aiming at the SIP in PPI prediction problem, SMOTE [52], PSPEL [53], RP-FFT [44], SPAR [26], and LocFuse [54] have put forward better solutions to the problem. To better assess the capabilities of SIPGCN, we compared it with these models.

As the evaluation parameters used by these methods are inconsistent, we chose the accuracy provided by all of them as the measurement index, and summarized the results obtained in the human and yeast data sets in Table 7. From Table 7, it is evident that SIPGCN achieved the highest prediction accuracy in the human dataset, which is 3.80% higher than the average accuracy of other methods. SIPGCN also achieved the best results in the yeast data set, with an average accuracy that was 10.90% higher than other methods and 3.83% higher than the second highest PSPEL method. The outcome of the comparison experiments indicates that SIPGCN has a better performance and can predict SIP more accurately than the previous models.

## 4. Discussion

In this work, we designed an effective SIP prediction model SIPGCN based on protein amino acid sequences, combined with a deep learning GCN and RF classifier. We first used the PSSM matrix to obtain the evolutionary message of the amino acids, then extracted their hidden feature distributions using the GCN algorithm, and finally utilized the RF classifier on the gold standard data sets to determine whether there were interrelationships between them. SIPGCN shows an optimal performance after comparison with different models and previous methods. These excellent results indicate that SIPGCN has the ability to accurately predict SIP and can provide new insights for wet experiments.

There are two reasons SIPGCN performs so well. Firstly, SIPGCN makes full use of the evolutionary message of protein amino acids, which provides an excellent solution for the characterization of the sequence information; secondly, the feature extraction ability of deep learning GCN is quite impressive, which can extract the hidden feature distribution in protein network nodes as much as possible and can represent it as a numerical vector. With the support of these two advantages, SIPGCN naturally has a powerful prediction capability.

However, there are some limitations of SIPGCN. For example, SIPGCN only uses the sequence information of proteins and does not utilize their physicochemical information or 3D structure information, which needs to be further explored. Additionally, although the deep learning GCN method has a strong feature extraction capability, it has a high complexity and a large number of hyperparameters. How to better tune these hyperparameters to achieve optimal performance and reduce their complexity needs to be further resolved. These limitations motivate us to continuously improve the method and measure the performance of SIPGCN with higher requirements.

## 5. Conclusions

Proteomics research has always occupied an important position in biology research, and protein self-interaction prediction studies are also progressing and break-throughs have been made. In this work, we designed an innovative model, SIPGCN, for predicting SIP based on deep learning. The model utilizes the evolutionary message of protein amino acids and mines their deep features using the GCN algorithm. On the gold standard data set, SIPGCN has demonstrated its excellent predictive power. SIPGCN has also exhibited an optimal performance in ablation experiments. The above described results demonstrate that SIPGCN can accurately predict proteins with self-interaction and can rapidly provide credible candidates for wet experiments.

## Figures and Tables

**Table 1:** The FFCV outcomes attained using SIPGCN in the human data set.

Testing Set	Acc.	Spe.	MCC	F1	AUC
1	93.53%	99.68%	49.63%	44.72%	0.6108
2	93.41%	99.84%	41.77%	33.62%	0.6198
3	92.78%	99.03%	37.20%	34.81%	0.5841
4	94.10%	99.85%	32.09%	22.64%	0.5422
5	94.42%	99.78%	54.36%	49.74%	0.6773
Average	93.65 ± 0.64%	99.64 ± 0.35%	43.01 ± 9.04%	37.11 ± 10.54%	0.6068 ± 0.0496

**Table 2:** The FFCV outcomes attained using SIPGCN in the yeast data set.

Testing Set	Acc.	Spe.	F1	MCC	AUC
1	91.32%	99.82%	44.33%	49.87%	0.6599
2	91.08%	98.84%	35.09%	37.04%	0.6413
3	90.35%	98.99%	41.75%	43.85%	0.6838
4	90.11%	98.72%	37.56%	39.07%	0.6181
5	90.60%	99.01%	33.14%	36.13%	0.6122
Average	90.69 ± 0.50%	99.08 ± 0.43%	38.37 ± 4.63%	41.19 ± 5.69%	0.6430 ± 0.0297

**Table 3:** The outcomes of different classifier models in the human data set.

Model	Testing Set	Acc.	Spe.	MCC	F1	AUC
ELM	1	86.88%	93.55%	14.33%	21.38%	58.32%
	2	86.99%	92.58%	11.95%	19.00%	56.66%
	3	88.26%	94.26%	16.77%	23.02%	56.21%
	4	86.62%	92.72%	11.27%	18.56%	55.46%
	5	87.21%	93.17%	14.56%	21.52%	54.59%
	Average	87.19 ± 0.63%	93.26 ± 0.68%	13.78 ± 2.21%	20.70 ± 1.87%	56.25 ± 1.40%
KNN	1	87.34%	93.81%	11.76%	18.52%	54.53%
	2	87.63%	93.49%	15.11%	21.82%	59.00%
	3	87.17%	93.00%	12.81%	19.78%	56.21%
	4	86.30%	92.65%	13.44%	20.93%	53.86%
	5	87.55%	93.62%	13.25%	19.96%	56.66%
	Average	87.20 ± 0.53%	93.31 ± 0.48%	13.27 ± 1.22%	20.20 ± 1.25%	56.05 ± 2.01%
SIPGCN	Average	93.65 ± 0.64%	99.64 ± 0.35%	43.01 ± 9.04%	37.11 ± 10.54%	60.68 ± 4.96%

**Table 4:** The outcomes of different classifier models in the yeast data set.

Model	Testing Set	Acc.	Spe.	MCC	F1	AUC
ELM	1	79.18%	87.01%	8.93%	20.80%	55.50%
	2	79.82%	85.93%	10.53%	21.32%	56.27%
	3	80.14%	86.47%	12.70%	23.53%	55.02%
	4	80.87%	86.96%	17.41%	27.88%	59.00%
	5	78.39%	86.04%	10.14%	22.48%	51.64%
	Average	79.68 ± 0.94%	86.48 ± 0.50%	11.94 ± 3.35%	23.20 ± 2.82%	55.49 ± 2.64%
KNN	1	82.32%	90.59%	12.60%	22.54%	59.12%
	2	83.44%	90.68%	10.92%	20.16%	52.72%
	3	81.75%	90.11%	12.21%	22.53%	54.78%
	4	82.88%	91.35%	11.35%	20.82%	53.99%
	5	83.94%	92.07%	9.94%	18.70%	54.18%
	Average	82.86 ± 0.87%	90.96 ± 0.76%	11.40 ± 1.06%	20.95 ± 1.64%	54.96 ± 2.44%
SIPGCN	Average	90.69 ± 0.50%	99.08 ± 0.43%	41.19 ± 5.69%	38.37 ± 4.63%	64.30 ± 2.97%

**Table 5:** Comparison of SIPGCN with an AC feature model in the human data set.

Model	Testing Set	Acc.	Spe.	MCC	F1	AUC
AC	1	84.12%	90.71%	5.05%	13.75%	49.99%
	2	83.94%	89.58%	7.97%	16.47%	57.12%
	3	83.22%	89.87%	6.17%	15.38%	52.51%
	4	85.04%	90.15%	9.46%	17.20%	55.81%
	5	85.23%	91.35%	7.97%	16.01%	55.79%
	Average	84.31 ± 0.82%	90.33 ± 0.71%	7.32 ± 1.72%	15.76 ± 1.30%	54.24 ± 2.93%
SIPGCN	Average	93.65 ± 0.64%	99.64 ± 0.35%	43.01 ± 9.04%	37.11 ± 10.54%	60.68 ± 4.96%

**Table 6:** Comparison of SIPGCN with the AC feature model in the yeast data set.

Model	Testing Set	Acc.	Spe.	MCC	F1	AUC
AC	1	78.62%	87.73%	0.88%	13.07%	52.37%
	2	78.62%	85.69%	7.51%	19.39%	54.44%
	3	80.63%	87.87%	10.10%	20.98%	58.29%
	4	79.02%	86.57%	6.37%	18.18%	55.90%
	5	80.16%	87.09%	10.04%	21.09%	55.84%
	Average	79.41 ± 0.93%	86.99 ± 0.90%	6.98 ± 3.77%	18.54 ± 3.29%	55.37 ± 2.17%
SIPGCN	Average	90.69 ± 0.50%	99.08 ± 0.43%	41.19 ± 5.69%	38.37 ± 4.63%	64.30 ± 2.97%

**Table 7:** Comparison of accuracy among SIPGCN and previous models in the human and yeast data sets.

Data Set	SIPGCN	SMOTE	PSPEL	RP-FFT	SPAR	LocFuse
*Human*	93.65%	91.68%	91.30%	93.54%	92.09%	80.66%
*Yeast*	90.69%	85.49%	86.86%	82.96%	76.96%	66.66%

## Data Availability

Data is contained within the article and Supplementary Material.

## Supplementary Materials

The following supporting information can be downloaded at: https://www.mdpi.com/article/10.3390/biomedicines10071543/s1. Table S1: Accuracy results of different hyperparameters generated by grid search method.
