# Supplementary material for: SIPGCN: A Novel Deep Learning Model for Predicting Self-Interacting Proteins from Sequence Information Using Graph Convolutional Networks
Source: Biomedicines. 2022 Jun 29;10(7):1543. doi: 10.3390/biomedicines10071543 (PMC9313220; doi:10.3390/biomedicines10071543)
Supplement: Supplementary file 1 [file biomedicines-10-01543-s001.zip › biomedicines-1765486-supplementary.pdf]

Table S1. Accuracy results of different hyperparameters generated by grid search method.

learning rate\hidden neurons	16	32	64	128	256	512	1024
0.02	86.43%	88.13%	89.73%	91.23%	93.53%	90.75%	90.15%
0.04	87.09%	88.71%	90.18%	91.41%	93.95%	91.31%	90.79%
0.06	87.78%	89.28%	90.78%	91.78%	94.13%	91.78%	91.21%
0.08	88.49%	89.67%	90.84%	92.17%	94.30%	93.10%	91.80%
0.10	88.51%	89.92%	91.15%	92.38%	94.42%	93.07%	91.62%
0.12	88.13%	89.37%	91.07%	92.26%	94.28%	93.00%	91.29%
0.14	87.84%	89.22%	90.46%	91.90%	94.10%	92.86%	91.01%
0.16	87.46%	88.73%	90.21%	91.52%	93.77%	92.27%	90.64%
0.18	86.97%	88.14%	89.85%	91.08%	93.27%	91.49%	90.19%
